# Fatty acid metabolism-related signature predicts survival in patients with clear cell renal carcinoma

**DOI:** 10.18632/aging.204433

**Published:** 2022-12-12

**Authors:** Rongjiang Wang, Junwen Shen, Yu Chen, Jianguo Gao, Jianxiang Yao

**Affiliations:** 1 The Department of Urology, The First Affiliated Hospital of Huzhou Normal College, Huzhou, Zhejiang 31300, China; 2 The Department of Urology, The First Hospital of Huzhou, Huzhou, Zhejiang 31300, China; 3 Huzhou Key Laboratory of Precise Diagnosis and Treatment of Urinary Tumors, Huzhou, Zhejiang 31300, China

**Keywords:** fatty acid metabolism, ccRCC, survival, signature, immune component

## Abstract

Objective: To explore fatty acid metabolism-related genes and signature, which could predict survival outcomes of clear cell renal carcinoma (ccRCC) patients.

Materials and Methods: Transcriptional and survival data of fatty acid genes in ccRCC patients were retrieved from UCSC Xena and Geo DataSets. We first performed Lasso Cox regression analysis to identify survival-related genes. These genes were then used to construct metabolic-related gene signature and risk score. Enrichment analysis and immune component and chemotherapy response prediction were also performed.

Results: In total, five survival-related genes were identified: *AGR2*, *HAO2*, *IGF2BP1*, *MCCD1* and *OLFM4* (*p* < 0.05). A series of survival value analyses revealed survival-related signature and risk score, including KM analysis (training set: *p* < 0.001; test set: *p* = 0.008). Four clinical indexes (T stage, N stage, M stage, and pathology) were positively correlated with risk score. Time-dependent ROC analysis yielded AUC value of 0.813. Immune landscape analysis revealed that risk score was strongly correlated with TAM score and cytotoxic score. Patients with high risk score and TAM score or cytotoxic score had the shortest survival time. Finally, inhibition of fatty acid metabolism in human ccRCC cell line produced corresponding changes in five genes, consistent with our preliminary results.

Conclusion: We identified five survival-related genes (*AGR2*, *HAO2*, *IGF2BP1*, *MCCD1* and *OLFM4*) in ccRCC patients. Our results also indicated that survival-related signature based on these genes is a potential robust prognostic biomarker for ccRCC in patients.

## INTRODUCTION

Self-sufficiency in growth, tissue invasion and metastasis are typical tumor characteristics [1]. To support these proliferative activities, tumor tissue must undergo metabolic reprogramming. A classic example of metabolic reprogramming was the Warburg’s effect in which glucose undergoes anaerobic glycolysis in tumor cells [2]. In recent years, several studies have shown that a few metabolic substrates, including glucose, lactic acid, fatty acid, and amino acid, are preferentially utilized for cancer growth, invasion and metastasis. Lipid metabolism has also been reported to perform a role in cancer metastasis. Previously understood as simple metabolic substrates, fatty acids are now known to influence lipid level in tissues, becoming a subject of much research interest [3].

Clear cell renal carcinoma is a urological malignant tumor with high incidence in the population. However, effective treatment for advanced renal clear carcinoma in patients is still lacking. Developing new molecular drug targets for treating advanced renal clear carcinoma would potentially improve survival outcomes of patients. Based on previous literature, key fatty acid proteins were reported to be strongly correlated with renal clear cell carcinoma’s growth, invasion and metastasis [4, 5]. Inhibition of fatty acid synthase was shown to activate P53 and STAT pathways, decreasing growth of renal cancer cells [6]. Moreover, fatty acid binding proteins (FABP) have been implicated in renal cell carcinogenesis [7].

However, fatty acid metabolism-related genes set associated with clear cell renal carcinoma in patients have not been systematically studied. In this study, we investigated key fatty acid-related protein and potential signature predicting survival of renal clear cell carcinoma patients to provide a basis for future research directions.

## MATERIALS AND METHODS

### Data source

Two clear cell renal carcinoma patients’ datasets with survival results were used in this study: TCGA-KIRC dataset, including RNA sequences and clinical phenotypes, downloaded from the UCSC Xena [8] and GSE29609 dataset, including expression matrix and survival results, downloaded from the GEO DataSets [9]. These two datasets were publicly available from the databases. We used TCGA-KIRC dataset as a training set to perform a series of survival analyses and identify a survival-related signature. In contrast, GSE29609 was designed as a test set for testing the signature’s accuracy and stability.

### Survival-related fatty acid genes

Based on a review of previous literature, 90 fatty acid-related genes were selected [10]. To find the survival value of each of the 90 fatty acid genes, we performed Lasso cox regression analysis on the TCGA-KIRC dataset. Subsequently, univariate and multivariate Cox regressions were performed on genes with survival value to find survival-related fatty acid genes.

### Signature of survival-related fatty acid genes

We predicted a new signature containing survival-related fatty acid genes. The following formula was used to calculate each sample risk score:


$$\text{Risk Score}=\sum^{\text{i}}(\text{Coefi}\times \text{ExpGenei})$$

“Coef” represents non-zero regression coefficients calculated with univariate Cox regression analysis. “ExpGene” represents expression values of genes from the prognostic risk score model. We calculated risk score level for TCGA-KIRC dataset’ patients and verified the survival value of the signature in both the training set and test set using KM analysis.

### Relationship between risk score and clinical features

First, using test set, we performed a time-dependent ROC analysis to predict risk score on 1-, 3- and 5-year survival results. Second, important clinical prognostic features, such as age and TMN stage, were compared in the training set using Cox regression analysis. R package “survival”, and “glmnt” were used for ROC and Cox regression analyses. We constructed a forest plot showing these results. In addition, prognostic characteristics with significantly different risk scores selected for further analysis. A nomogram of overall survival for clear cell renal carcinoma patients in the training set with risk scores and other important clinical prognostic indexes was drawn.

### Enrichment analysis for low- and high-risk score patient groups

Patients included in the training set were divided into two groups: high risk-score group and low risk-score group. R package ‘limma’ was used to identify differentially expressed genes between two patient groups. Genes with FDR <0.05 were considered statistically significant. Three functional enrichment analyses were performed on differentially expressed genes for the two groups: GO analysis [11], KEGG analysis [12], and Gene set variation analysis (GSVA) [13]. R packages “cluster Profile” and “GSVA” were used for enrichment analysis. Based on enrichment analysis results, we identified candidate different functions between the high-score group and low-score group patients.

### Immune-related characteristics in low- and high-risk score groups

We performed multi-dimension immune-related analysis on the two groups of patients. First, we used cibersort website (https://cibersort.stanford.edu/) to analyze the immune component of the two patients groups and divided patients into immune subtypes [14]. Second, we used R package “GSVA” to analyze immune-related score. Third, we explored immune status of the two groups using R package “estimate”. Fourth, the TAM score and Cytotoxic score, important prognostic indexes generated using R package “GSVA”, were compared between the two patient groups. Finally, we compared the expression levels of two important immune checkpoint genes: *CTLA4* and *PDL1*.

### Chemotherapy drug sensitivity analysis

Ridge regression model was constructed with R package “pRRophetic” and “oncoPredict” using the remaining data. Drug sensitivity (IC50) was chosen as an outcome index. Chemotherapeutic response was predicted using tumor genes expression and drug sensitivity data of cell lines from the cancer genome project (CGP) and drug sensitivity in cancer (GDSC) [15].

### Verification of survival-related fatty acid genes in cell lines

Increasing the expression level of fatty acid synthase (FASN) was positively correlated with aggressive cell proliferation, migration, apoptosis, and lipid droplet formation. It was also shown to regulate metabolic disorders associated with clear cell renal carcinoma. Researchers also proved that pharmacological inhibitor of FANS could suppress the growth and invasiveness of clear cell renal carcinoma. Therefore, we used two human clear cell renal carcinoma cell line (caki-1 and 786-0 cell lines) from authenticated cell cultures of the Chinese national collection. We down-regulated the expression of FANS in caik-1 cells by the way of siRNA virus and exposed FASN inhibitorC75 (HY-12364, MedChemExpress, China) with two cell line (caki-1 and 786-0). We verified the expression level of survival-related fatty acid genes with quantitative real-time polymerase chain reaction (Q-PCR) [16].

### Data availability statement

The original data presented in this study are included in the article. All data were retrieved from public databases.

## RESULTS

### Identity of survival-related fatty acid genes

**Figure 1:**
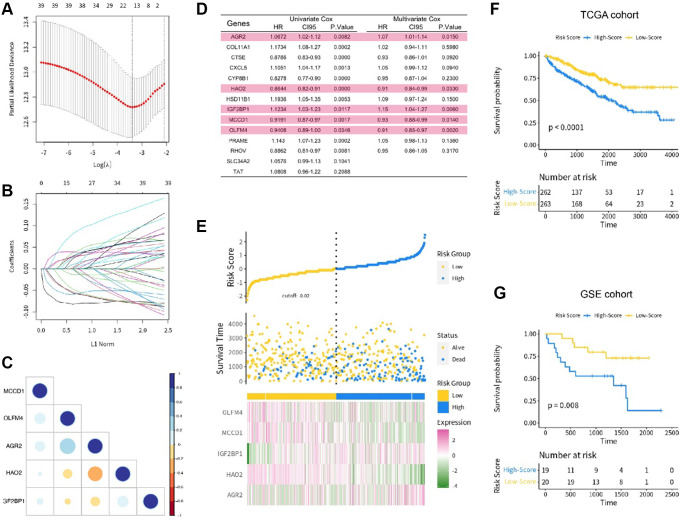
(**A**, **B**) The lasso regression analysis; (**C**) The correlation of 5 key genes; (**D**) The univariate and multivariate COX regression analysis; (**E**) The risk score distribution in the training set; (**F**) The KM analysis in the TCGA cohort; (**G**) The KM analysis in the GSE cohort.

We downloaded 90 fatty acid genes, 525 patient clinical records and RNA sequences from the TCGA-KIRC dataset (the training set) and survival results of 39 renal cancer patients and the matrix of genes’ expression from the GSE29609 (the test set). We performed Lasso regression analysis on the training set for all fatty acid genes (Figure 1A, 1B). The results showed that only 14 genes had survival value for renal cancer patients. After univariate and multivariate Cox regression analyses (Figure 1D), only five survival-related genes (*AGR2, HAO2, IGF2BP1, MCCD1* and *OLFM4*) were significantly different to have the statistical value (*p* < 0.05). The correlation among the five genes was also established in the Figure 1C.

### Risk score construction and survival value verification

The risk score was calculated using the following formula:

Risk score = (0.0726303) × *AGR2* + (−0.17735540) × *HAO2* + (0.18400723) × *IGF2BP1* + (−0.08972776) × *MCCD1* + (−0.08952658) × *OLFM4*.

**Figure 2:**
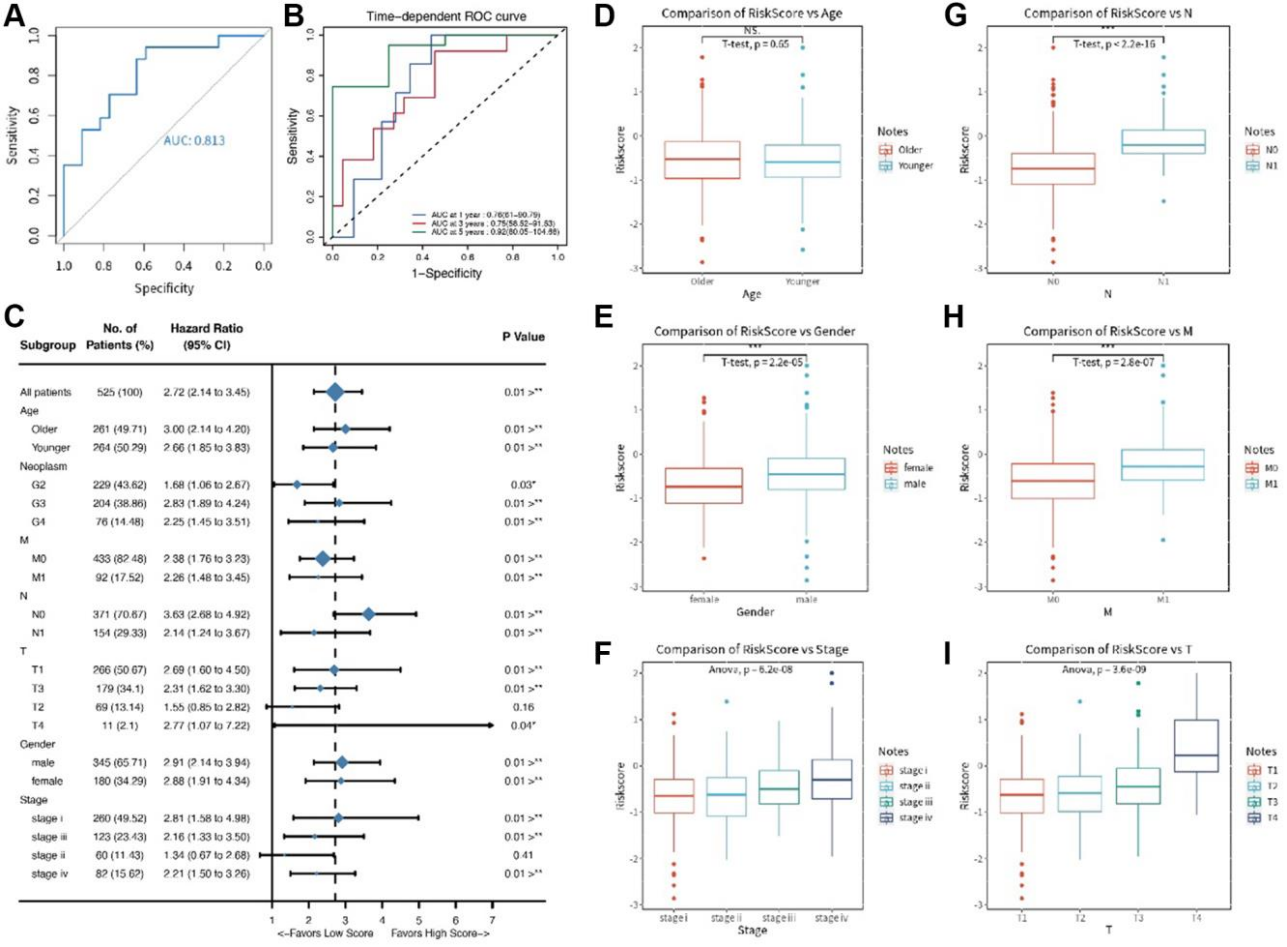
(**A**, **B**) The ROC time dependent analysis; (**C**) The forest plot for clinical features in the training set; (**D**–**I**) The comparative results for age, gender, T stage, N stage, M stage, and pathology stage.

The patients in the training set were divided into low- and high-risk score groups based on their risk score level. We also presented the distribution of risk score in the training set (Figure 1E). To explore the survival value of each risk score, we tested it on both training and test sets using KM analysis. The results showed that renal cancer patients with high risk score had shorter survival time for both the training set (*p* < 0.001, Figure 1F) and test set (*p* = 0.008, Figure 1G). We also used time-dependent ROC curve to analyze the risk score in the test set. The AUC value of risk score was 0.813, with satisfactory results for ROC curves (Figure 2A, 2B).

### Relationship between risk score and clinical features

**Figure 3:**
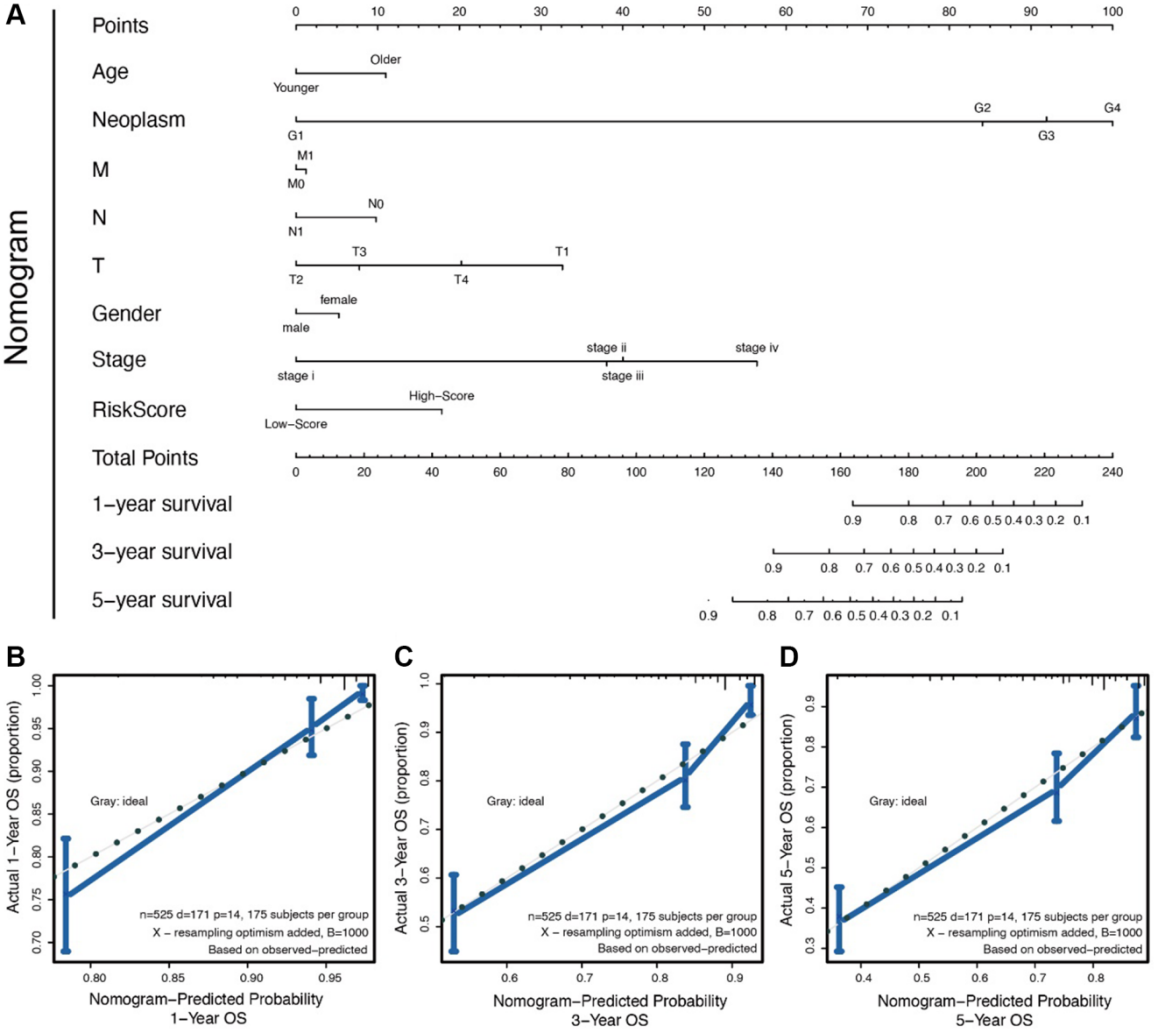
(**A**) The nomogram for all the clinical features; (**B**–**D**) The nomogram to predict probability 1-/3-/5- year OS.

We compared clinical features (like TMN stages) in the training set using Cox regression analysis. The results showed that six clinical features (age, T stage, N stage, M stage, gender and pathology stage) had significant survival value (*p* < 0.05, Figure 2C) and were presented in a forest plot. A further separate comparison of these six indexes between the two groups showed that patients with high-risk score had worse T stage, N stage, M stage, and pathology stage (*P* < 0.001) than low-risk score patients (Figure 2D–2I). A nomogram containing several clinical features and risk score was drawn predicting 1-, 3- and 5-year overall survival (Figure 3A–3D).

### Enrichment analysis for low- and high-risk score patient groups

**Figure 4:**
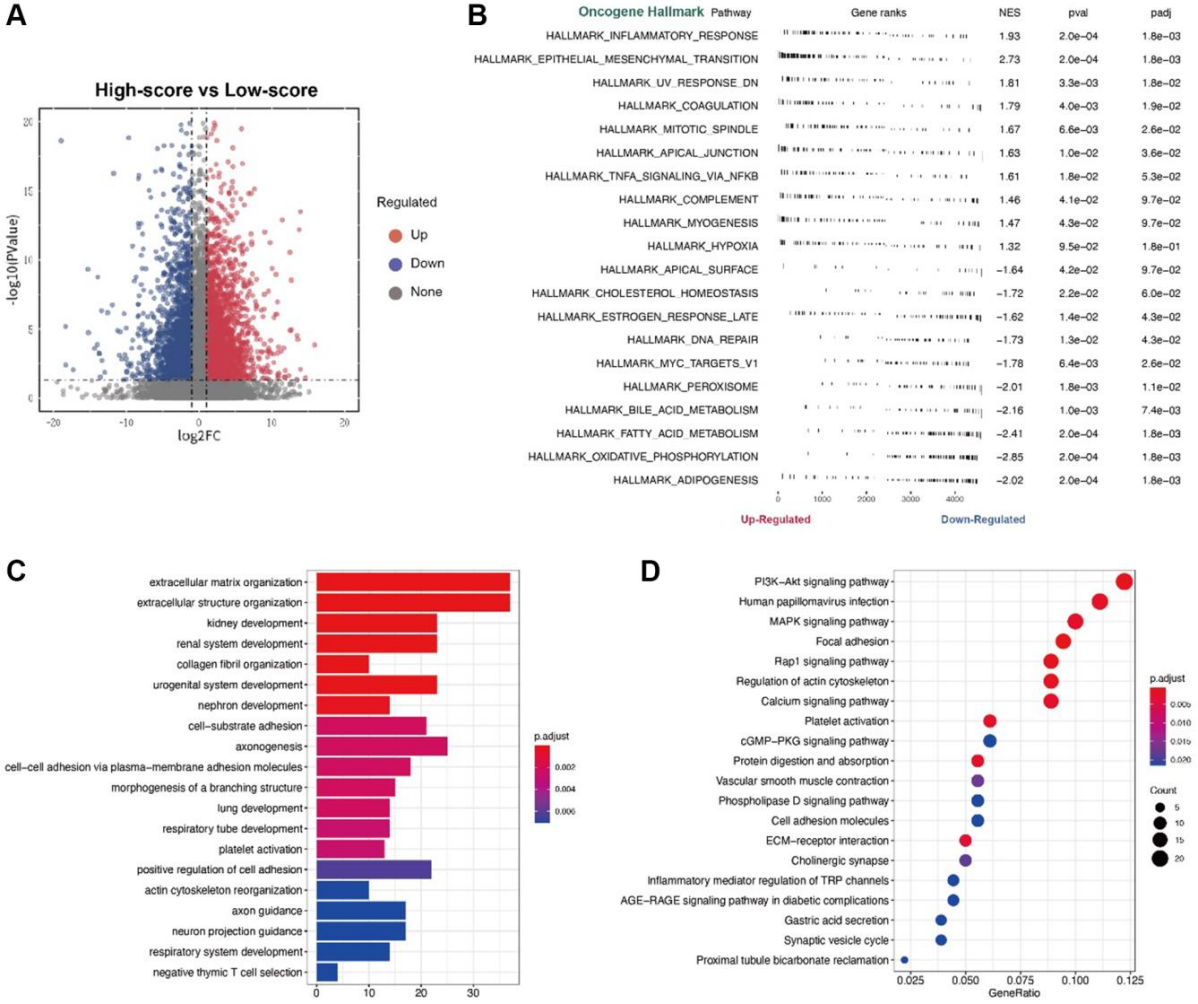
(**A**) The volcano plot for the different expression level genes; (**B**) The GSVA analysis; (**C**) The GO analysis; (**D**) The KEGG analysis.

Using R package “limma”, we identified differentially expressed genes, which were represent in a volcano plot (Figure 4A). Three forms of functional enrichment analysis were used to determine potential function of risk score in two groups of renal cancer patients. First, GO analysis showed that functions of extracellular matrix organization, extracellular structure organization, and urogenital system development would be activated in high-risk score patients (Figure 4C). In contrast, the functions of negative thymic T cell selection and actin cytoskeleton reorganization were inhibited in high-risk score patients. Second, KEGG analysis showed that pathways of PIK3, papillomavirus infection, and MAPK signaling would be activated in high-risk score patients (Figure 4D). Third, GSVA analysis ultimately found that a high-risk score was strongly correlated with inflammatory response and epithelial mesenchymal response in patients (Figure 4B).

### Immune-related characteristics in low- and high-risk score groups and chemotherapy drug sensitivity analysis

**Figure 5:**
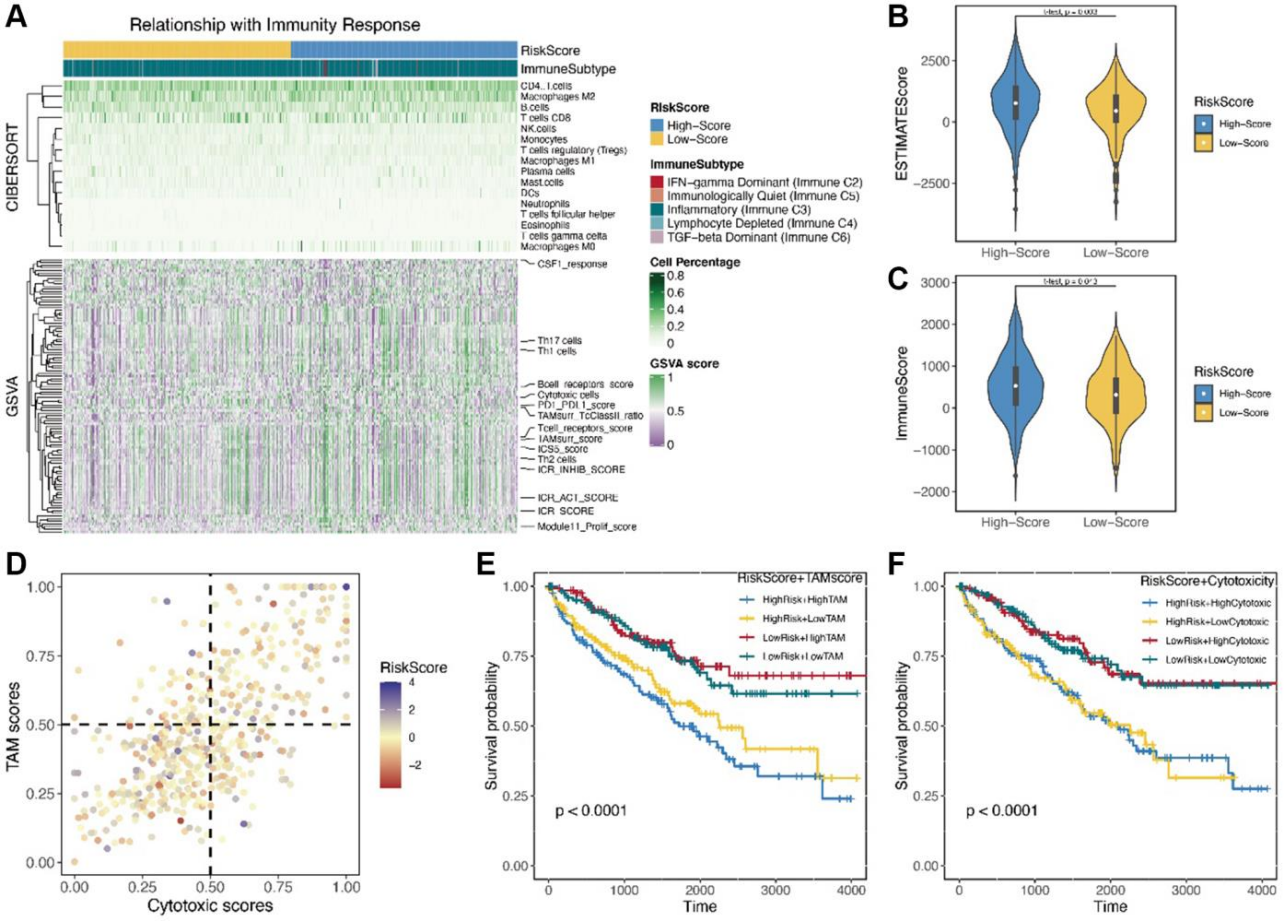
(**A**) The landscape of immunity response by the cibersort and GSVA; (**B, C**) The immune score and estimate score by the R package “estimate”; (**D**) The correlation between the TAM score and cytotoxic score; (**E**, **F**) The KM analysis of TAM score and cytotoxic score.

**Figure 6:**
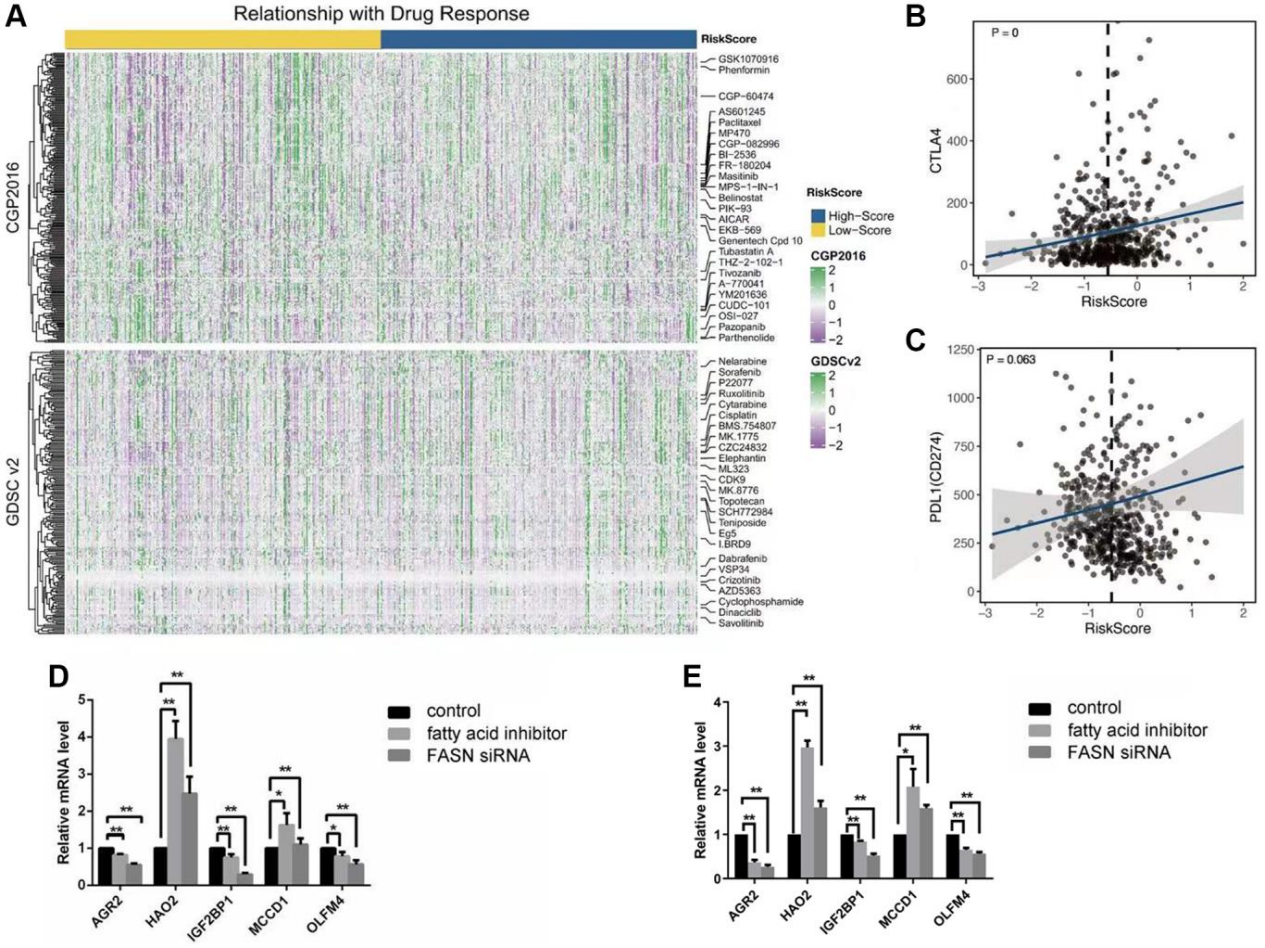
(**A**) The landscape of drug response by the CGP and GDSC; (**B**, **C**) The correlation between the risk score and the expression level of CTLA4 and PDL1; (**D**, **E**) The results of 5 key genes by the test of Q-PCR among three groups: control group; fatty acid inhibitor group, and the FASN siRNA group, caki-1 cell line (**D**); 786-0 cell line (**E**).

Using cibersort website, we identified immune components of two patient groups. However, differences between main immune cells could not be identified by scanning whole immune landscapes of the two groups (Figure 5A). However, high-risk score patient group were more likely to exhibit immune insensitivity subtypes, such as immune C2 and C5, than low-risk score patients. Subsequently, we used R package “estimate” to analyze the estimate score and immune score (Figure 5B, 5C). The results showed that immune scores were significantly different between the two patient groups (*p* = 0.043). Scanning the immune scores with R package “GSVA” revealed a strong correlation of TAM score and cytotoxic score were with risk score. TAM score and cytotoxic score have been reported to have a prognostic value for many cancers. Here, we analyzed the two scores for renal cancer patients. The results showed a strong positive survival value of TAM score and cytotoxic score in renal cancer patients (Figure 5D). KM analysis further revealed that patients with high-risk score and TAM score or cytotoxic score had poorer survival results than low-risk score patients (*p* < 0.001, Figure 5E, 5F). We further compared the correlation of risk score with two important immune therapy genes: *PDL1* and *CTLA4*. The results showed a strong positive correlation between risk score and expression level of *PDL1* or *CTLA4* (Figure 6B, 6C). Finally, we performed chemotherapy drug sensitivity analysis on CGP and GDSC platforms. The landscape of drug sensitivity analysis was presented in Figure 6A.

### Verification of survival-related fatty acid genes in caki-1 cell line

We verified survival related fatty acid genes in caki-1 cell and 786-0 cell (two human renal clear cell carcinoma cell lines) in two ways: down-regulating FASN expression and exposure to pharmacological inhibitor of FASN. We measured expression levels of five survival-related fatty acid genes. The results showed that three genes (*AGR2, IGF2BP1* and *OLFM4*) had been inhibited whereas two genes (*HAO2* and *MCCD1*) had been activated in both two ways (Figure 6D, 6E). These changes suggested that the five genes regulated fatty acid synthase function in renal cancer patients. Therefore, the five-gene signature could be used to predict survival outcomes of renal cancer patients.

## DISCUSSION

Lipid metabolism has attracted significant attention in cancer research in recent years because it provides a novel connection between blockers of lipid metabolism and inhibitors of tumor growth and invasion [17]. FASN regulates fatty acid metabolism and plays a critical role in the growth and tissue invasion by many cancers [18, 19]. We found a few studies providing deep analysis of the role of FASN in clear cell renal carcinoma development. The study by Xu and team found that *FASN* expression was positively correlated with aggressive cell proliferation, migration, apoptosis and lipid droplet formation and regulated metabolic disorders in clear cell renal carcinoma microenvironment [6]. Further, using a pharmacological inhibitor of fatty acid synthase has been shown to suppress growth and invasiveness of renal cancer cells [20].

With increasing research evidence for the role of fatty acids in clear cell renal carcinoma development in patients, analysis of fatty acid genes was warranted in the present study. With this purpose, we conducted a series of analyses to identify these genes. In total, we identified five survival-related fatty acid genes implicated in ccRCC. Consistent with these results, *HAO2* was reported to inhibit malignancy of clear cell renal cell carcinoma by promoting lipid catabolic process [21]. In addition, *AGR2* and *IGF2BP1* promoted tumorigenesis by accelerating hypoxia state in renal cell carcinoma cell line [22–23].

However, no direct metabolic function could be identified for the other three genes (*MCCD1,* and *OLFM4*) in research. We used two ways to inhibit fatty acid metabolism in clear cell renal carcinoma cell line. The five survival-related genes exhibited corresponding expression variation upon Q-PCR test. Consistent with these results, previous studies showed that inhibition of fatty acid metabolism in a cell line using the two methods could suppress cancer cell line growth or invasion. Our results illustrated that the expression levels of the five genes not only reflected the fatty acid state of clear cell renal carcinoma patients, but were also strongly correlated with the survival of clear cell renal carcinoma patients.

A metabolism-related signature, based on the five survival related fatty acid genes, was proven as a reliable prognostic model for clear cell renal carcinoma patients. Firstly, using KM analysis, the survival value of the signature was confirmed in both the training and test sets. Our results revealed a significant statistical association (training set: *p* < 0.001; test set: *p* = 0.008). Secondly, six clinical indexes with significant survival value were found in clear cell renal carcinoma patients. Further, the risk scores of the signature in four clinical indexes were positively correlated. Thirdly, ROC analysis revealed a high AUC value of 0.813 for the risk score of the signature. In addition, a five-year time-dependent ROC analysis revealed a satisfied AUC value of 0.92. Because the metabolism-related signature had a high AUC value, the sensitivity and specificity of this signature were higher than those of signatures reported by a few previous studies in renal cancer patients [24–25].

Metabolic reprogramming usually transforms immune ability and immune components in cancer tissues [2]. Interdisciplinary research examining metabolic reprogramming and immune function is increasingly pursued in cancer studies. We used three methods to identify the risk score, immune ability and immune component: cibersort website, estimate and GSVA. We found no significant differences in the immune component between low-risk and high-risk score groups using cibersort. In contrast, using estimate, we found stronger immune ability in the high-risk score group than in the low-risk score group. In addition, we analyzed different immune-related scores to determine TAM score and cytotoxic score. Our results showed that patients with high-risk score and TAM score or cytotoxic score had lower survival time than those with low-risk score. Recent studies on immune cells have found a correlation between TAM cells were and cancer progression and anti-immunotherapy [26]. So far, few researchers have studied the connection between the fatty acid metabolism and percentage or function of TAM cells in the cancer patients. Our results suggested a potential connection, providing a novel direction for future studies. Cytotoxic score, which has not been clearly defined in literature, was positively correlated with risk score or TAM score in clear cell renal carcinoma patients. Therefore, cytotoxic score provided a new approach to predicting survival outcomes of clear cell renal carcinoma patients.

Studies examining fatty acid metabolism, a major metabolism reprogramming mechanism in cancer patients, have elicited much research interest in the topic in past several years. Whereas *FASN* has been a subject of intense research, the other fatty acid-related genes have not been studied for their role in cancer. The multifarious functions of fatty acid proteins in cancer provide another research direction that needs further exploration.

## CONCLUSION

We identified five survival-related genes (*AGR2, HAO2, IGF2BP1, MCCD1* and *OLFM4*) in clear cell renal carcinoma patients. The survival signature based on these genes was proved to have a significant prognostic value for clear cell renal carcinoma in patients.

## References

[1] Hanahan D, Weinberg RA. Hallmarks of cancer: the next generation. Cell. 2011; 144:646–74. 10.1016/j.cell.2011.02.013 21376230

[2] Xia L, Oyang L, Lin J, Tan S, Han Y, Wu N, Yi P, Tang L, Pan Q, Rao S, Liang J, Tang Y, Su M, et al. The cancer metabolic reprogramming and immune response. Mol Cancer. 2021; 20:28. 10.1186/s12943-021-01316-8 33546704 PMC7863491

[3] Merino Salvador M, Gómez de Cedrón M, Moreno Rubio J, Falagán Martínez S, Sánchez Martínez R, Casado E, Ramírez de Molina A, Sereno M. Lipid metabolism and lung cancer. Crit Rev Oncol Hematol. 2017; 112:31–40. 10.1016/j.critrevonc.2017.02.001 28325263

[4] Bobulescu IA, Pop LM, Mani C, Turner K, Rivera C, Khatoon S, Kairamkonda S, Hannan R, Palle K. Renal Lipid Metabolism Abnormalities in Obesity and Clear Cell Renal Cell Carcinoma. Metabolites. 2021; 11:608. 10.3390/metabo11090608 34564424 PMC8470169

[5] Lai Y, Tang F, Huang Y, He C, Chen C, Zhao J, Wu W, He Z. The tumour microenvironment and metabolism in renal cell carcinoma targeted or immune therapy. J Cell Physiol. 2021; 236:1616–27. 10.1002/jcp.29969 32783202

[6] Xu W, Hu X, Anwaier A, Wang J, Liu W, Tian X, Zhu W, Ma C, Wan F, Shi G, Qu YY, Zhang H, Ye D. Fatty Acid Synthase Correlates With Prognosis-Related Abdominal Adipose Distribution and Metabolic Disorders of Clear Cell Renal Cell Carcinoma. Front Mol Biosci. 2021; 7:610229. 10.3389/fmolb.2020.610229 33569391 PMC7868388

[7] Wu G, Zhang Z, Tang Q, Liu L, Liu W, Li Q, Wang Q. Study of FABP's interactome and detecting new molecular targets in clear cell renal cell carcinoma. J Cell Physiol. 2020; 235:3776–89. 10.1002/jcp.29272 31602654 PMC13482663

[8] Wang S, Xiong Y, Zhao L, Gu K, Li Y, Zhao F, Li J, Wang M, Wang H, Tao Z, Wu T, Zheng Y, Li X, Liu XS. UCSCXenaShiny: An R/CRAN Package for Interactive Analysis of UCSC Xena Data. Bioinformatics. 2021; 38:527–9. 10.1093/bioinformatics/btab561 34323947 PMC8723150

[9] Barrett T, Wilhite SE, Ledoux P, Evangelista C, Kim IF, Tomashevsky M, Marshall KA, Phillippy KH, Sherman PM, Holko M, Yefanov A, Lee H, Zhang N, et al. NCBI GEO: archive for functional genomics data sets--update. Nucleic Acids Res. 2013; 41:D991–5. 10.1093/nar/gks1193 23193258 PMC3531084

[10] Ding C, Shan Z, Li M, Chen H, Li X, Jin Z. Characterization of the fatty acid metabolism in colorectal cancer to guide clinical therapy. Mol Ther Oncolytics. 2021; 20:532–44. 10.1016/j.omto.2021.02.010 33738339 PMC7941088

[11] Gene Ontology Consortium. Gene Ontology Consortium: going forward. Nucleic Acids Res. 2015; 43:D1049–56. 10.1093/nar/gku1179 25428369 PMC4383973

[12] Kanehisa M, Furumichi M, Tanabe M, Sato Y, Morishima K. KEGG: new perspectives on genomes, pathways, diseases and drugs. Nucleic Acids Res. 2017; 45:D353–61. 10.1093/nar/gkw1092 27899662 PMC5210567

[13] Hänzelmann S, Castelo R, Guinney J. GSVA: gene set variation analysis for microarray and RNA-seq data. BMC Bioinformatics. 2013; 14:7. 10.1186/1471-2105-14-7 23323831 PMC3618321

[14] Chen B, Khodadoust MS, Liu CL, Newman AM, Alizadeh AA. Profiling Tumor Infiltrating Immune Cells with CIBERSORT. Methods Mol Biol. 2018; 1711:243–59. 10.1007/978-1-4939-7493-1_12 29344893 PMC5895181

[15] Yang W, Soares J, Greninger P, Edelman EJ, Lightfoot H, Forbes S, Bindal N, Beare D, Smith JA, Thompson IR, Ramaswamy S, Futreal PA, Haber DA, et al. Genomics of Drug Sensitivity in Cancer (GDSC): a resource for therapeutic biomarker discovery in cancer cells. Nucleic Acids Res. 2013; 41:D955–61. 10.1093/nar/gks1111 23180760 PMC3531057

[16] Singh C, Roy-Chowdhuri S. Quantitative Real-Time PCR: Recent Advances. Methods Mol Biol. 2016; 1392:161–76. 10.1007/978-1-4939-3360-0_15 26843055

[17] Menendez JA, Lupu R. Fatty acid synthase and the lipogenic phenotype in cancer pathogenesis. Nat Rev Cancer. 2007; 7:763–77. 10.1038/nrc2222 17882277

[18] Menendez JA, Lupu R. Fatty acid synthase (FASN) as a therapeutic target in breast cancer. Expert Opin Ther Targets. 2017; 21:1001–16. 10.1080/14728222.2017.1381087 28922023

[19] Schroeder B, Vander Steen T, Espinoza I, Venkatapoorna CMK, Hu Z, Silva FM, Regan K, Cuyàs E, Meng XW, Verdura S, Arbusà A, Schneider PA, Flatten KS, et al. Fatty acid synthase (FASN) regulates the mitochondrial priming of cancer cells. Cell Death Dis. 2021; 12:977. 10.1038/s41419-021-04262-x 34675185 PMC8531299

[20] Horiguchi A, Asano T, Asano T, Ito K, Sumitomo M, Hayakawa M. Pharmacological inhibitor of fatty acid synthase suppresses growth and invasiveness of renal cancer cells. J Urol. 2008; 180:729–36. 10.1016/j.juro.2008.03.186 18555493

[21] Xiao W, Wang X, Wang T, Chen B, Xing J. HAO2 inhibits malignancy of clear cell renal cell carcinoma by promoting lipid catabolic process. J Cell Physiol. 2019; 234:23005–16. 10.1002/jcp.28861 31127626

[22] Pajdzik K, Wilamowski M, Żurawek D, Stopa KB, Nodzyński M, Kalita A, Jura J. Anterior gradient 2 promotes tumorigenesis through upregulation of CCAAT-enhancer binding protein beta and hypoxia-inducible factor-2α and subsequent secretion of interleukin-6, interleukin-8, and vascular endothelial growth factor in the Caki-1 clear cell renal cell carcinoma cell line. IUBMB Life. 2020; 72:1807–18. 10.1002/iub.2331 32593213

[23] Jiang Y, Zhang H, Li W, Yan Y, Yao X, Gu W. LINC01426 contributes to clear cell renal cell carcinoma progression by modulating CTBP1/miR-423-5p/FOXM1 axis via interacting with IGF2BP1. J Cell Physiol. 2021; 236:427–39. 10.1002/jcp.29871 32583425

[24] Shen J, Wang R, Chen Y, Fang Z, Tang J, Yao J, Ling Y, Zhang L, Zhang X. An Immune-Related Signature Predicted Survival in Patients With Kidney Papillary Cell Carcinoma. Front Oncol. 2021; 11:670047. 10.3389/fonc.2021.670047 34164341 PMC8215362

[25] Hong Y, Lin M, Ou D, Huang Z, Shen P. A novel ferroptosis-related 12-gene signature predicts clinical prognosis and reveals immune relevancy in clear cell renal cell carcinoma. BMC Cancer. 2021; 21:831. 10.1186/s12885-021-08559-0 34281531 PMC8290606

[26] Ngambenjawong C, Gustafson HH, Pun SH. Progress in tumor-associated macrophage (TAM)-targeted therapeutics. Adv Drug Deliv Rev. 2017; 114:206–21. 10.1016/j.addr.2017.04.010 28449873 PMC5581987

